# Bibliographical

**Published:** 1879-02

**Authors:** 


### Editorial.

### BIBLIOGRAPHICAL.

PHYSIOLOGY. Preliminary course of Lectures. By James T. Whittaker, A. M., M. D., Professor of Physiology and Clinical Medicine in the Medical College of Ohio; Lecturer on Clinical Medicine at the Good Samaritan Hospital; Member of the Cincinnati Academy of Medicine, and of the Cincinnati Society of Natural History. On the Influence of Physiology upon Practice; on the Conservation of force; on the Origin of Life, and the Evolution of its Forms, and Protoplasm, Bone, Muscle, Nerve and Blood. Illustrated.

In the preface the author says: "I have endeavored in the delivery of these lectures to put within the reach and comprehension of the first course student the foundation facts and principles upon which the stately edifice of physiology is built."

In this little work of about two hundred and eighty pages, are set forth in clear, terse and sharply defined manner, the fundamental principles of physiology, so that they may be readily comprehended by the student. It is not only valuable to the student but to the practitioner as well, as a work of ready reference to salient points.

It occupies a position that has heretofore been vacant, at least so far as any single work is concerned. It is a work valuable to every specialist as well as to the general practitioner. I do not attempt to review the work, but trust that every one will procure and review it for himself.

